# Socio-culturally responsive medical professionalism and ethics education: A curriculum co-creation approach

**DOI:** 10.1080/10872981.2024.2303209

**Published:** 2024-01-09

**Authors:** Nurfarahin Nasri, Wenwen Xu, Khairul Azhar Jamaludin, Nurfaradilla Mohamad Nasri

**Affiliations:** a Faculty of Education, Universiti Kebangsaan Malaysia, Bangi, Selangor, Malaysia; b Faculty of Aviation, Chengdu College of University of Electronic Science and Technology of China, Chengdu, China

**Keywords:** Curriculum, curriculum co-creation, curriculum development, socio-culturally responsive curriculum, medical professionalism and ethics education

## Abstract

Medical professionalism and ethics (MPE) are critical components influencing how medical practitioners provide patients with the highest standard of care. As a result, a structured attempt has been undertaken to enhance the content and teaching delivery of the medical professionalism and ethics education (MPEE) in the undergraduate medical curriculum. Guided by Vygotsky’s sociocultural learning theory, Harre and Van Langenhove’s positioning theory and Taba’s principles of curriculum development, a curriculum co-creation project was organized with the aim of developing a socio-culturally responsive MPEE. A total of fifteen medical students agreed to participate in the project where they co-created MPE curriculum with a medical educator over the course of three months. Upon completion of the project, a co-created, socio-culturally responsive MPE curriculum was presented. The thematic analysis revealed positive changes in the participants’ attitudes, skills, and behaviors towards co-creating the MPE curriculum. They also reported feeling a sense of fulfilment after having a transformative experience as curriculum co-creators and after receiving positive feedback from the faculty, staff, and other students on the co-created MPE curriculum. The project’s success demonstrates the importance of curriculum co-creation as a strategy to promote co-creation efforts among students and educators in developing a socio-culturally responsive curriculum. The project’s framework and practical recommendations can be adopted by other medical educators and faculties to encourage students’ participation and their role on curriculum development using the co-creation approach.

## Introduction

Ethics and professionalism are critical in the medical profession. For the younger generation of medical practitioners, the changing environment of modern medicine in the twenty-first century presents considerable challenges and opportunities. Emerging issues which include but are not limited to increasing diversity of patients, growing alternative medicine, evolving technological breakthroughs and persisting global pandemic will continue to raise ethical challenges or dilemma that must be urgently addressed [1]. Specific to Malaysia, where there are over 60 different ethnic groups living together and an annual influx of migrants from various parts of the world, the country’s growing population dynamics place pressure on medical practitioners to be cognizant of religious and cultural differences that may challenge their professional or ethical stance. Numerous steps are undertaken to ensure that patients from different cultural backgrounds do not receive inferior medical care, yet less emphasis is given to reshaping medical education to ensure that it can produce medical practitioners who are socio-culturally responsive. As a result, there is an urgency for medical faculties in Malaysia to continuously improve medical education curriculum especially in the area of medical professionalism and ethics education (MPEE) [2].

Meanwhile, curriculum co-creation is growing in prominence as a pedagogical method that fosters a democratic learning environment where educators and students can more effectively understand one another’s viewpoints on teaching and learning [3]. Curriculum co-creation advances culturally relevant forms of teaching and learning that in turn support the development of socio-culturally conscious students by drawing on the educator’s teaching experience as well as the learners’ funds of knowledge (FOK) [4,5]. Additionally, a co-created curriculum allows students to participate in decision-making that determines important facets of teaching and learning, hence supporting inclusive, high-quality education [6]. In this study, we define curriculum co-creation as an innovative pedagogical approach to develop a socio-culturally responsive MPEE in the Malaysian context.

## Our Malaysian context

The Malaysian Medical Council (MMC) through its division of the Medical Education Committee (MEC) jointly work with the Malaysian Qualifications Agency (MQA) to facilitate the quality of medical education in the country by designing quality assurance documents. The revised version of ‘Standards for Undergraduate Medical Education 2022’ is designed in line with the current Malaysian Qualifications Framework 2.0 (MQF), Codes of Practice of Programme Accreditation (COPPA), and Guidelines to Good Practices. Medical ethics, according to the standard document refers to dealing with ‘moral issues in medical practice such as values, rights and responsibilities related to physician behaviour and decision making’ [7]. MPEE is also one of the 18 core competencies that medical graduates must master during their preclinical years and clinical trainings. Table 1 shows the MPEE learning topics and competency area which are outlined in the standard document.

**Table 1.** Learning topics and competency area for MPEE.

Ethics	Professionalism
Principles of Biomedical Ethics	Respect
Good Medical Practice	Responsibility & Accountability
Code of Professional Conduct	Excellence and Scholarship
Consent	Honour and Integrity
Confidentiality	Altruism
Research Ethics	Leadership and Team work
Clinical Ethics	Cultural Competency and Managing Diversity
Ethical Issues at the End of Life	Compassion and Empathy
Ethical Issues in Paediatrics	Confidentiality
Ethical Issues in Obstetrics and Gynaecology	
Ethical Issues in Organ Donation	
MMC Ethical Guidelines	

A previous study conducted at one of the local medical faculties claimed that despite standardizing learning topics in MPEE, medical students still face difficulties in demonstrating sufficient level of ethical and professionalism skills [8]. The students are still oblivious of the idea of ethics and professionalism and request for MPEE teaching to be more specific, relevant, and challenging [9]. From a pedagogical point of view, there is also no known consensus on the best successful approach of teaching MPEE, and lecturing remains the most widely used method [10]. As a result, a high percentage of medical students has been reported to feel indifferent towards MPEE and are unsure of the implication of ethics and professionalism towards their career [11].

These underlying issues inform us the gap between what the MPE curriculum intends to achieve and what the students perceive about its relevance and applicability in their socio-culturally diverse workplace setting. Along the same line, we argue that while there are plenty of studies investigating the perspectives of medical students on medical ethics and professionalism, yet none offers an explicit understanding on how these perspectives can be successfully utilized into developing a socio-culturally responsive MPEE. Below, we outline our project to address this problem, project outcomes, and lessons learned.

## Our innovative approach

Guided by Vygotsky’s socio-cultural learning theory [12], we implemented a curriculum co-creation project at University X involving fifteen third-year medical students and a medical educator to enhance the content and teaching delivery of MPEE, in particular to develop a socio-culturally responsive MPEE. More specifically, we highlighted the importance to tap into students’ FOK regarding socio-cultural experiences and perspectives during curriculum co-creation sessions to integrate relevant learning topics into MPEE [13]. These tenets are consistent with the curriculum co-creation approach which focuses on building interaction, relationship and negotiation between educators and learners, while being willing to embrace larger social structures. Additionally, the project adopts Harre and Van Langenhove’s positioning theory, where people may position themselves or be positioned by others within a particular social context [14]. This makes the theory relevant as it helps to understand the roles that educators and students play in co-creating the curriculum. We also took careful consideration on ensuring the project aligns with Taba’s principles of curriculum development, namely flexibility and responsibility sharing, curriculum relevance or coherence and holistic student development [15,16].

The students were selected using convenience sampling since, at the time, they were the only group of students registering for the course. All participants submitted the Informed Consent to indicate their voluntary participation and their agreement for subject privacy and confidentiality. It is crucial to note that the students had no prior exposure, experience or training in curriculum co-creation while the medical educator had a three-year experience of co-creating curriculum. However, before starting the project, the students were given a short briefing about curriculum co-creation, specifically regarding their expected roles and ways in which they could contribute to the process. The students were advised about their authority to assume various roles such as co-designers or consultants during the curriculum co-creation process.

This project comprised 26 hours of group discussion sessions to co-create the MPE curriculum and highlight socio-culturally responsive topics, and reflective sessions to gain perspectives of the project outcomes. More specifically, we conducted a two-hour discussion (2 × 11 weeks) to ensure close student-educator engagement, active negotiations and critical decision-making could take place while ensuring the program remained feasible for the medical educator. As an example of how students participated in curriculum co-creation, the students negotiated for the integration of several learning units and added topics that they considered to be pertinent, such the different cultural practices in end-of-life care, cultural traditions in confinement and cultural views of organ donation. The students also proposed that it was vital to discuss some topics from a medico-legal perspective. This resulted in a shared understanding of the importance of having certain course contents taught by qualified lawyers specialized in medical law.

Moreover, we arranged a two-hour focus group discussions (FGDs) during the mid and final week of the project to explore students’ and medical educator’s experiences of co-creating curriculum. The semi-structured discussions were conducted in English, audio-recorded and analyzed via hybrid thematic analysis [17]. We started the analysis by getting familiar with the data and transcribing the recordings manually. We continued rereading the transcripts for multiple times and defined the codes line-by-line. Next, we eliminated any codes that were unclear and grouped codes with similar meanings together under one possible theme. Finally, the data-driven themes were constantly compared in terms of their consistency within and across the entire data set. We also made constant comparisons between the findings from previous studies and new, unexpected data to identify commonalities and discrepancies.

Table 2 illustrates the procedures employed in this project.

**Table 2.** Adaptation of Taba’s principles, Harre and Van Langenhove’s positioning theory and Vygotsky’s sociocultural learning theory on our project framework.

Week 1 – Week 5	Responsibility-sharing between medical educator and students	Setting co-creation environment, describing roles and expectations Diagnosing individual and group learning needs Negotiating learning objectives/goals across knowledge domain, attitude domain and skill domain Selecting contents that carry socio-culturally relevance Organizing contents into subtopics or learning units	Co-creation tools: Role playing Discussion building Scaffolding tools: Goal mapping Brainstorming FOK tools: Background survey Heritage short essays Positions: Co-designers Consultants
Week 6		First FGD session	
Week 7 – Week 12		Selecting learning experience through pedagogical exploration Organizing learning experience based on pedagogical choice Designing assessment and ways to implement Aligning objectives, content, pedagogy and assessment Refining the co-created, socio-culturally responsive MPE framework	
Week 13		Second FGD session	

## Project outcomes

We organized the project outcomes into two deliverables, namely the co-created MPE curriculum from the discussion sessions and the qualitative findings from the FGDs. The new MPE curriculum contains ten specific topics that the co-creation group found to be socio-culturally responsive for medical practitioners in Malaysia. The new curriculum was evaluated by an expert panel (MPE educator, curriculum specialist, culturally responsive educationist) and received collective agreement for its novelty and adaptability in actual classroom settings. Table 3 shows the simplified framework of the co-created MPE curriculum on selected learning topics.

**Table 3.** A fraction of the socio-culturally responsive MPE curriculum designed via curriculum co-creation.

	Learning units			
	Palliative care	Post-partum care	Organ donation	Miscellaneous
Added topics of discussion	Cultural pain differences	Sociocultural beliefs and confinement (*berpantang*) practices	Individual right vs social responsibility	Artificial intelligence in healthcare
	Religious concept of grief/dying	The ‘silent’ mother	Religious views on organ donation	Who are you in social media?
	End-of-life cultural practices	Traditional and complementary medicine use	Legislation issue	Patient-doctor relationship in social media

Based on the FGDs findings, two overarching themes emerged. The first theme describes how the project fostered positive attitudes, skills and behaviors among the participants. The students shared having favorable attitudes and attained important skills including decision-making, critical judgment and negotiating. Whereas the medical educator reported better acquisition of classroom management skills and showed more receptivity to student input. The second theme addresses the participants’ sense of fulfilment and how all students had a powerful experience playing their role as curriculum co-creators as they were able to select, research, add and integrate topics into the curriculum. Meanwhile, the medical educator reflected back on the positive response provided by the faculty, other staffs and students regarding the co-created curriculum which prompted her to continue using this co-creation approach in her teaching course.

## Lessons learned

The curriculum co-creation project continues to be a reference guide for other medical educators at the faculty to integrate the approach into their teaching courses. Despite the project’s success, we believe that there is no one-size-fits-all strategy in implementing curriculum co-creation, possibly due to its complicated nature that changes the student-educator power dynamic [18]. Despite not having a standardized means to this approach, we strongly recommend others to adhere to key defining cores of what constitutes an effective curriculum co-creation project. We suggest to start co-creating curriculum with a smaller group of students as this allowed better control of the overall process and the outcomes of the approach. This, however, does not imply that the medical educator is in full control of the approach; rather, it accentuates the value of responsibility sharing. We also recommend to co-create curriculum with willing students and receptive medical educators as it lessens the impact of power dynamic through a mutual, respectful relationship between both parties.

Furthermore, we suggest medical educators to be vigilant, adaptable, and critical in identifying possible hindrance that could inhibit the approach or opportunity that might be advantageous to them. In our case, such complexity was exacerbated by the already packed medical curriculum and the lack of student engagement. The majority of the project consisted of the discussion sessions, and some finished beyond the allotted time for different reasons, including the failure to reach consensus on particular issues or arrive to a conclusion. It was also discovered that the students used the discussion sessions as an opportunity to unwind from their clinical works which had left them feeling exhausted and preferring not to engage in conversation. As such, we decided against pressuring the students to actively participate in those sessions’ discussions; instead, we opted to wait and watch. The result was ideal since the students gradually and consciously started taking on more active roles throughout the discussion. In the future, we would welcome any administrative support with the MPEE schedule planning to ensure both medical educators and students are prepared to contribute and exchange ideas in a constructive, stable environment.

Given that the nature of curriculum co-creation necessitates the active participation of each member, it is recommended that a typology for the co-creation process be developed to facilitate this intricate approach. This is to help decide the nature of this approach which includes its direction, types of involvement, focus, participants, expected outcomes and recompense, with the aim of promoting an organic co-creation process. Furthermore, this typology allows for the broadening of the scope of this process, extending beyond the confines of student involvement. The inclusion of additional experts who possess relevant expertise is crucial, albeit within a regulated environment to prevent the emergence of dominance, discussions and reflections that are led solely by experts. As we advance our project, we are continuously updating strategies to raise awareness about curriculum co-creation, incorporate transdisciplinary expertise, enhance assessment method and expand it to a larger co-creation group.

## Conclusion

In conclusion, this study has provided a clear direction for expanding the traditional way of developing curriculum, which emphasizes needs rather than involving the target users as part of the curriculum design and development. Given the nature of curriculum co-creation, which demands active discussion and negotiation, the design of the curriculum will therefore be customized to specific needs and settings of the course and the institution.
